# Exploring molecular superfluidity in hydrogen clusters

**DOI:** 10.1126/sciadv.adu1093

**Published:** 2025-02-21

**Authors:** Hatsuki Otani, Susumu Kuma, Shinichi Miura, Majd Mustafa, Jeff C. W. Lee, Pavle Djuricanin, Takamasa Momose

**Affiliations:** ^1^ Department of Chemistry, The University of British Columbia, Vancouver, British Columbia V6T 1Z1, Canada.; ^2^ Atomic, Molecular and Optical Physics Laboratory, RIKEN, Wako, Saitama 351-0198, Japan.; ^3^ Faculty of Mathematics and Physics, Kanazawa University, Kakuma, Kanazawa 920-1192, Japan.; ^4^ TRIUMF, Vancouver, British Columbia V6T 2A3, Canada.

## Abstract

Molecular hydrogen (H_2_) has long been predicted to exhibit superfluidity—a state of zero viscosity—at extremely low temperatures. However, its existence remains under debate despite several experimental reports. In this study, we investigated the infrared transitions of methane embedded in clusters of parahydrogen molecules at 0.4 K using high-resolution helium nanodroplet spectroscopy. Our results revealed fully quantized rotational states of methane with minimal interference from surrounding H_2_ molecules, enabling precise determination of the rotational constant for each hydrogen cluster. The cluster-size dependence of the determined rotational constant aligns with behavior predicted by path-integral Monte Carlo simulations, indicating that more than 60% of the hydrogen molecules in the clusters participate in quantum bosonic exchanges, a characteristic feature of superfluidity. This work provides strong experimental evidence for the existence of a superfluid phase of molecular hydrogen at 0.4 K, representing a major step forward in understanding quantum behaviors in molecular systems.

## Teaser

Experiments reveal superfluidity in hydrogen clusters at 0.4 K, highlighting unique quantum behaviors of molecular hydrogen.

## INTRODUCTION

Superfluidity is a remarkable state of matter characterized by the frictionless flow of a fluid, exhibiting properties that are fundamentally distinct from those of ordinary liquids. This unique phenomenon, occurring only at extremely low temperatures, has been observed in bulk helium and in clusters of ultracold atoms, capturing the attention of scientists for decades. Molecular hydrogen (H_2_), the simplest and lightest molecule, has also been predicted to become superfluid in the liquid state at low temperatures (*1*). However, despite its potential, the existence of a superfluid phase in molecular hydrogen remains a subject of ongoing debate.

H_2_ is a spinless composite boson similar to the ^4^He atom but with a lighter mass. By considering intermolecular interactions between hydrogen molecules, the superfluid transition temperature *T*_s_ is predicted to be 1 to 2 K (*2*–*7*). However, as the predicted transition temperature is lower than the bulk freezing point of hydrogen at 13.8 K, finite-size clusters have been proposed as a potential pathway to maintain the liquid phase down to *T*_s_ (*8*). Because *T*_s_ depends on the degeneracy *g* as *g*^−2/3^, the *para* nuclear spin (*I* = 0) species, *p*H_2_, is considered to be a more preferable candidate for achieving superfluidity than its counterpart *ortho* (*I* = 0) species, *o*H_2_.

The superfluidity of both atomic (He) and molecular (H_2_) clusters has been investigated using rotational spectroscopy of an embedded probe molecule, which is the microscopic manifestation of Andronikashvili’s experiment (*9*). The superfluidity thus examined is called microscopic superfluidity in contrast to the macroscopic superfluidity in bulk liquids. Early studies of helium droplets at 0.4 K revealed that embedded molecules exhibit quantized free rotations, a phenomenon attributed to the superfluid nature of helium droplets. A decrease in rotational constants was observed, reflecting the influence of the surrounding He atoms, which respond adiabatically to the molecular rotation (*10*, *11*).

Microscopic superfluidity of molecular hydrogen clusters has been reported as the disappearance of the *Q*-branch of OCS-(*p*H_2_)*_N_* clusters (*N* = 13 to 16) at 0.15 K in ^3^He/^4^He mixed droplets (*12*, *13*). However, this observation remains a subject of debate, partly because a similar *Q*-branch disappearance has been reported in HCN-(HD)*_N_* clusters (*14*), where HD is a fermion.

The change in the rotational constant of a probe molecule inside clusters has been used as an alternative probe of microscopic superfluidity (*15*, *16*). In molecular beam experiments, the free rotation of linear molecules in H_2_ clusters at temperatures around 1 K has been reported (*17*, *18*). These studies revealed that the rotational constant initially decreases with increasing cluster size, but begins to increase beyond a certain cluster size. This turnaround was called the onset of superfluidity, wherein the surrounding H_2_ clusters become decoupled from the rotation of the chromophore molecule due to enhanced superfluidity. A comprehensive review summarizing recent advances in this field is available in (*19*).

A recent theoretical study on pure (*p*H_2_)*_N_* cluster revealed large superfluid fractions in clusters with *N* ≤ 16, while in larger clusters, superfluidity is quenched especially at the magic numbers 26, 29, 32, and 37 below 1 K (*6*, *20*). The suppression of superfluidity in *p*H_2_ clusters has been observed in *p*H_2_-He mixed clusters doped with CO_2_, attributed to strong *p*H_2_-CO_2_ intermolecular interactions (*21*). Similar phenomena were also reported in (*22*).

Methane (CH_4_) is a spherical-top molecule with no electric dipole moment, characterized by a fast rotor with a large rotational constant. It exhibits very weak intermolecular interactions with *p*H_2_ [93 cm^−1^ (*23*) compared with 202 cm^−1^ for OCS (*24*)], making CH_4_ an ideal probe that minimally perturbs the superfluid environment. Previously, we have studied CH_4_-(*p*H_2_)*_N_* clusters in helium droplets via the ν_3_ stretching transition of CH_4_ (*25*). Because the linewidth of the ν_3_ vibrational transition was broad due to vibrational relaxation to lower-frequency modes, the cluster size dependence on the rotational constant could not be clearly resolved (*25*).

In this study, we investigated the ν_4_ rovibrational transition of CH_4_-(*p*H_2_)*_N_* in helium droplets at 0.4 K. Given that the ν_4_ bending mode is the lowest vibrationally excited state of CH_4_, it does not undergo linewidth broadening due to vibrational relaxation. Consequently, the linewidth remains exceptionally narrow, recorded at 65 MHz in helium droplets (*26*). This narrow linewidth makes the ν_4_ transition particularly suitable for exploring the superfluidity of *p*H_2_ clusters by examining the dependence of the rotational constant on cluster size.

## RESULTS

**Fig. 1.**
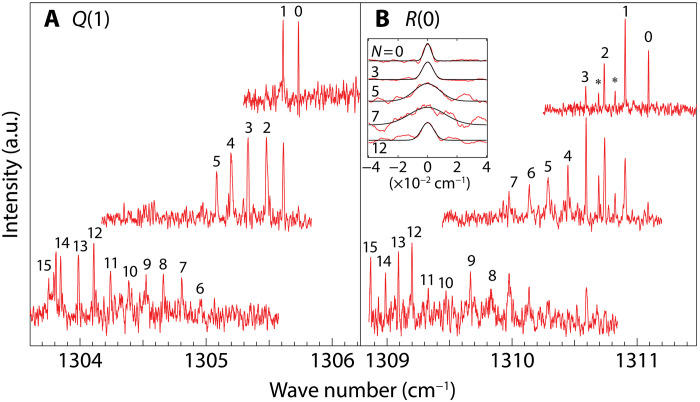
Rotationally resolved spectra of CH_4_-(*p*H_2_)*_N_* clusters. The rotationally resolved ν_4_ spectra of CH_4_-(*p*H_2_)*_N_* clusters with 0 ≤ *N* ≤ 15 in helium droplets. (**A**) *Q*(1) (*J* = 1 ← 1) rotational branch. (**B**) *R*(0) (*J* = 1 ← 0) rotational branch. Each peak is labeled with *N*, while peaks marked with ∗ are attributed to residual *o*H_2_ bearing clusters. The pressure of *p*H_2_ in the second pickup cell was increased progressively from the top to the bottom traces. Inset in (B) shows magnified line profiles of selected *R*(0) peaks relative to their peak positions, with Gaussian fitted curves displayed as black traces.

Figure 1 shows high-resolution depletion spectra of the ν_4_ transition of CH_4_-(*p*H_2_)*_N_* inside helium droplets. Because of the nuclear spin modifications, both the *J* = 0 and *J* = 1 rotational states of CH_4_ are occupied in helium droplets (*26*). Among the transitions satisfying the selection rule Δ*J* = 0 and ±1 for polyatomic molecules, the left panel shows the *Q*(1) (*J* = 1 ← 1) rotational transitions of CH_4_, whereas the right panel shows the *R*(0) (*J* = 1 ← 0) transitions. Other transitions, such as *R*(1) and *P*(1), were also observed. However, their analysis is more challenging due to asymmetric line shapes (*26*). Because the lowest-*J* transitions provide sufficient information for determining the rotational constants, this study focuses solely on the *Q*(1) and *R*(0) transitions. The top trace in each panel was taken at low *p*H_2_ pressure in the second pickup cell (see Materials and Methods and fig. S5), while the middle and bottom traces were recorded at medium and higher *p*H_2_ pressures, respectively. The highest wave number peak, labeled as 0 in the top trace, corresponds to the transition of CH_4_ in pure helium droplets, as reported previously (*26*). Increasing the *p*H_2_ pressure revealed additional sharp peaks at lower wave numbers with nearly constant spectral spacing. These peaks are attributed to the ν_4_ rotation-vibration transition of CH_4_-(*p*H_2_)*_N_* with different cluster size *N*. The cluster size assignments were based on the rotation-vibration analysis described below. The narrow linewidth of each peak enabled precise extraction of the cluster size dependence, in stark contrast to the previous ν_3_ band study (*25*).

The vibrational band origin ${\tilde{\nu }}_{0}$ and the rotational constant $\tilde{B}$, in units of cm^−1^, for each cluster size *N* were determined from the observed peak wave numbers of the *Q*(1) and *R*(0) transitions $[{\tilde{\nu }}_{Q\left(1\right)}$ and ${\tilde{\nu }}_{R\left(0\right)}$, respectively] based on the following equations (*27*)

$${\tilde{\nu }}_{Q\left(1\right)}={\tilde{\nu }}_{0}-2\tilde{B}\zeta$$ (1)

and

$${\tilde{\nu }}_{R\left(0\right)}={\tilde{\nu }}_{0}+2\tilde{B}\left(1-2\zeta \right)$$ (2)

The Coriolis coupling constant ζ was fixed to the gas phase value (*28*) of 0.46575 as before (*26*, *27*, *29*, *30*), and the same rotational constant $\tilde{B}$ was assumed for the ground and excited vibrational states.

**Fig. 2.**
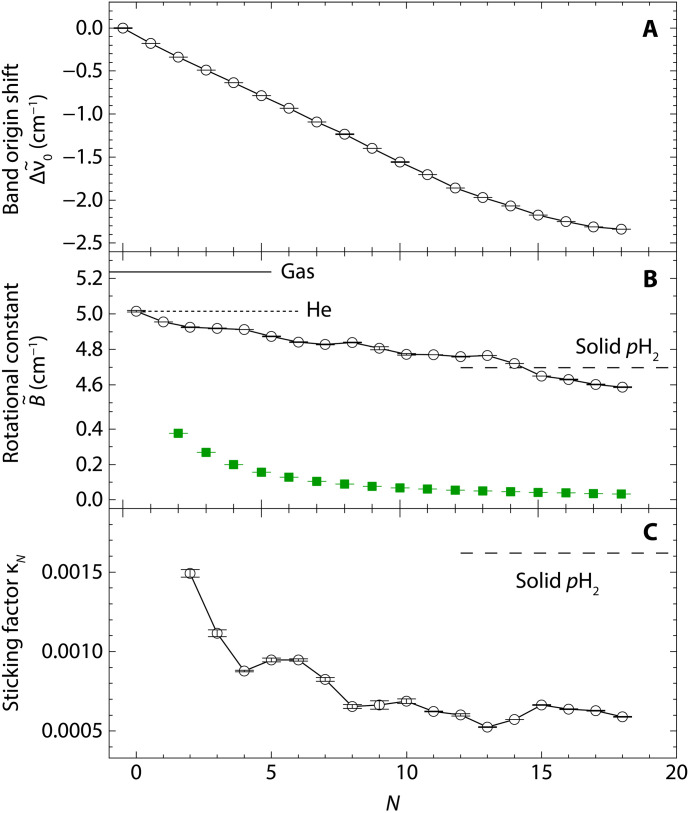
Spectral shift, rotational constant, and sticking factor of CH_4_-(*p*H_2_)*_N_* clusters. (**A**) Spectral shift of the vibrational band origin ${\tilde{\nu }}_{0}$ of CH_4_ in CH_4_-(*p*H_2_)*_N_* inside helium droplets relative to that of CH_4_ in helium droplets (*N* = 0) at 1310.49 cm^−1^. (**B**) Rotational constant $\tilde{B}$ of CH_4_-(*p*H_2_)*_N_* inside helium droplets as a function of *N*. The experimentally obtained rotational constant for each cluster size is shown by a black open circle with an error bar (1σ). The green filled squares represent the calculated rotational constants for rigid CH_4_-(*p*H_2_)*_N_* based on (*p*H_2_)*_N_* densities of PIMC simulations. The rotational constants of CH_4_ in the gas phase, pure helium droplets, and solid parahydrogen are depicted by a solid line, a dotted line, and a broken line, respectively. (**C**) The *N* dependence of the sticking factor κ*_N_* defined in Eq. 3, directly evaluated using the observed rotational constants and PIMC (*p*H_2_)*_N_* densities, assuming no superfluid component in the (*p*H_2_)*_N_* clusters. The dashed line indicates the solid value, κ_solid_.

The values of ${\tilde{\nu }}_{0}$ and $\tilde{B}$ for each *N* evaluated using Eqs. 1 and 2 are depicted in Fig. 2 (A and B, respectively). The smooth decrease in ${\tilde{\nu }}_{0}$ with increasing *N*, as shown in Fig. 2A (−0.153 cm^−1^ per molecule = −4.6 GHz per molecule for *N* ≤ 12), supports the cluster size assignments presented in Fig. 1. The rotational constants $\tilde{B}$ in Fig. 2B also exhibits gradual decrease as *N* increases. In helium droplets, the rotational constant of CH_4_ without any *p*H_2_ (*N* = 0) is 5% smaller than its value in the gas phase (*26*, *27*). The rotational constant of the CH_4_-(*p*H_2_)*_N_* clusters experiences a 13% reduction at *N* = 18.

The rotational constants $\tilde{B}$ in units of cm^−1^ are inversely proportional to the moment of inertia, I in SI units, expressed as $\tilde{B}$ = *h* × 10^−2^/(8π^2^*cI*), or $\tilde{B}$ = $16.85763/\bar{I}$, where $\bar{I}$ is the moment of inertia in units of uÅ^2^ (u, unified atomic mass unit). The moment of inertia of CH_4_-(*p*H_2_)*_N_* clusters, *I_N_*, can be calculated as the sum of the moment of inertia of CH_4_ ($I_{CH_{4}}$) and that of the surrounding (*p*H_2_)*_N_* clusters ($I_{pH_{2},N}$). However, because CH_4_ is a nearly spherical molecule with a large rotational constant, the surrounding (*p*H_2_)*_N_* clusters may not fully follow the rapid rotation of the central CH_4_ molecule. This phenomenon, known as rotational decoupling, is well established: Fast rotors, i.e., molecules with large rotational constants such as CH_4_ and NH_3_, can rotate freely in helium droplets, retaining the rotational constants similar to their gas-phase values (*26*, *31*).

The rotational decoupling is also evident in the case of (*p*H_2_)*_N_* clusters surrounding CH_4_. Notably, the observed rotational constants are substantially larger than those calculated under the assumption that the surrounding H_2_ molecules rotate in perfect synchrony with CH_4_, as shown in Fig. 2B. Here, the value of $I_{pH_{2},N}$ was evaluated using the density distribution of *p*H_2_ around CH_4_ obtained from the path-integral Monte Carlo (PIMC) simulation, which are discussed later. This discrepancy suggests a reduced effective contribution of $I_{pH_{2},N}$ to the total moment of inertia of CH_4_-(*p*H_2_)*_N_*, $I_{\mathrm{tot},N}.$ Consequently, $I_{\mathrm{tot},N}$ can be described by the following equation

$$I_{\mathrm{tot},N}=I_{CH_{4}}+\kappa_{N}I_{pH_{2},N}$$ (3)

The parameter κ*_N_* (where 1 ≥ κ*_N_* ≥ 0) represents the degree of contribution of the surrounding (*p*H_2_)*_N_* clusters to $I_{\mathrm{tot},N}$. This contribution reduction primarily arises from the relative slip of the (*p*H_2_)*_N_* clusters with respect to the rotational motion of CH_4_ (*32*). Hereafter, we refer to the parameter κ*_N_* as the sticking factor. A value of κ*_N_* = 1 corresponds to a case with no slip, whereas κ*_N_* = 0 indicates a case of perfect slip.

The ultimate example of a perfect slip case is observed in CH_4_ isolated in solid parahydrogen, where the surrounding *p*H_2_ molecules remain entirely stationary relative to the rotational motion of CH_4_. The average of the rotational constants of CH_4_ in solid parahydrogen in the ground and ν_4_ excited states is reported to be 4.69 cm^−1^ (*33*), corresponding to the moment of inertia of 3.59 uÅ^2^. This represents an increase in the moment of inertia of 0.38 uÅ^2^ compared to the gas phase value of 3.22 uÅ^2^ (*28*). Assuming the presence of the 12 nearest-neighbor molecules in the hexagonal close-packed (*hcp*) structure with a lattice constant of 3.78 Å, the sticking factor in the solid can be calculated as κ_solid_ = 0.00162. While CH_4_ in solid parahydrogen represents a perfect slip case, the sticking factor is not zero. This nonzero value is attributed to the minor lattice oscillations of the surrounding H_2_ molecules induced by the rotational motion of CH_4_. These oscillations are a consequence of slight asymmetry in the *J* = 1 rotational state wave function of CH_4_. These small lattice oscillations contribute to the kinetic energy of the CH_4_-(*p*H_2_)*_N_* clusters (*34*), resulting in a nonzero contribution to the moment of inertia.

The model discussed in (*34*) demonstrates that the increase in the moment of inertia caused by the surrounding H_2_ molecules is given by $\Delta I=\frac{1}{2}{\left(\frac{n\delta }{R}\right)}^{2}I_{H_{2},N=12}$. Here, *R* = 3.78 Å is the distance to the nearest-neighbor H_2_ molecules, δ is the mean amplitude of the lattice oscillations induced by the rotation of CH_4_, *n* = 2 is the asymmetry factor of the *J* = 1 rotational wave function, and $I_{H_{2},N=12}$ is the total moment of inertia of the 12 nearest-neighbor H_2_ molecules in an *hcp* lattice. This model suggests that the moment of inertia of the nearest-neighbor hydrogen molecules, $I_{H_{2},N=12}$, remains the essential quantity for describing changes in the moment of inertia under the perfect slipping condition. In addition, the parameter κ_solid_ is expected to have a small value due to the small amplitude δ of each hydrogen molecule’s oscillation near its equilibrium position relative to the intermolecular distance *R*.

An amplitude of δ ∼ 0.1 Å provides a reasonable explanation for the observed reduction in the rotational constant of CH_4_ in solid hydrogen, corresponding to a sticking factor of κ_solid_ = 0.00162. This value represents the physically acceptable lower bound for the sticking factor in Eq. 3, as any additional movement of the surrounding hydrogen molecules from their equilibrium positions would further increase the moment of inertia. The contribution of H_2_ molecules beyond the nearest neighbor is considered negligible due to the small amplitudes of lattice vibrations associated with methane rotation.

Figure 2C illustrates the sticking factors calculated using Eq. 3 based on the rotational constants of CH_4_-(*p*H_2_)*_N_* observed in helium droplets. The density distributions of (*p*H_2_)*_N_* obtained from the PIMC simulation were used to evaluate the values of $I_{pH_{2},N}$ whose radial distributions for *N* = 2 to 18 are provided in the Supplementary Materials. In our experiments, each CH_4_-(*p*H_2_)*_N_* cluster was surrounded by a superfluid He droplet. Because of the nonzero normal component of the superfluid He at 0.4 K, the moment of inertia of CH_4_ in the droplet is slightly larger than that of free CH_4_. To maintain a smooth relationship in the total moment of inertia, $I_{\mathrm{tot},N}$, from *N* = 0 to 18, we used the moment of inertia of CH_4_ in He droplets, $I_{CH_{4}}$, in Eq. 3 instead of its value in the gas phase. Using $I_{CH_{4}}$ from He droplets partially accounts for the influence of the surrounding He droplets on any cluster size of CH_4_-(*p*H_2_)*_N_*. However, as *N* increases, the effect of the surrounding He droplets would change due to the presence of (*p*H_2_)*_N_* clusters. This effect is implicitly included in the sticking factor in our analysis.

The derived sticking factors in helium droplets are much smaller than that of solid, κ_solid_, as shown in Fig. 2C. This result appears physically inconsistent, as intuitively, the sticking factor in helium droplets should be at least greater than κ_solid_ given that a portion of (*p*H_2_)*_N_* cluster might follow the rotational motion of the central CH_4_. The inconsistency likely arises from the fact that the superfluid fraction of the (*p*H_2_)*_N_* clusters was not accounted for in the above analysis.

Earlier PIMC simulations for CH_4_ in (*p*H_2_)*_N_* clusters demonstrated that a substantial fraction of the (*p*H_2_)*_N_* cluster exhibits superfluid behavior in smaller clusters (*35*). This phenomenon is expected to reduce the effective moment of inertia of the (*p*H_2_)*_N_* cluster, as the superfluid components are anticipated to contribute negligibly to the moment of inertia of the entire cluster.

**Fig. 3.**
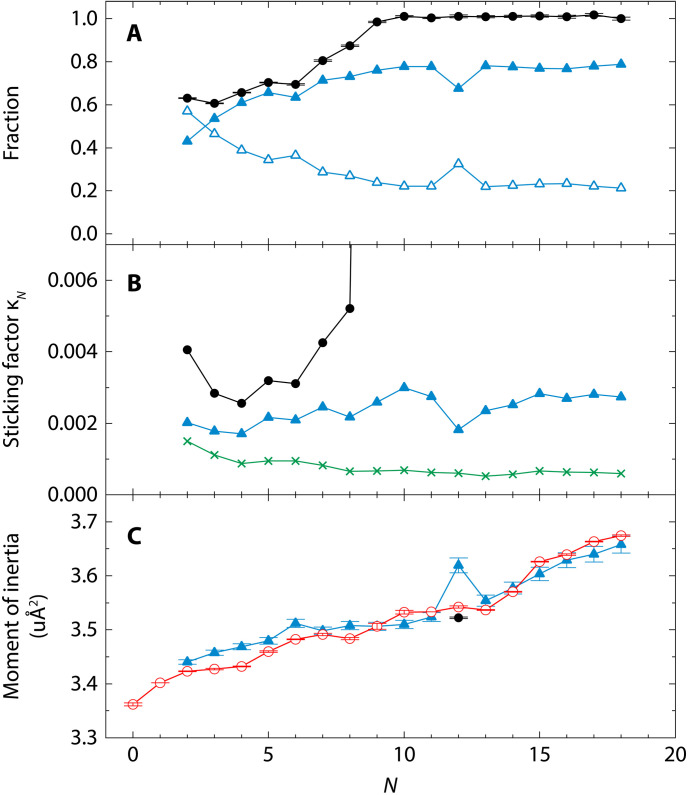
Microscopic superfluidity and sticking factor of (*p*H_2_)*_N_* clusters. (**A**) The fraction of superfluid component (black filled circles) in (*p*H_2_)*_N_* and that of the monomer component (blue open triangles) $\mu_{N}^{M}$, obtained from PIMC simulations. The blue filled triangles represent 1 − $\mu_{N}^{M}$, indicating the fraction of (*p*H_2_)*_N_* that participates in bosonic exchanges. (**B**) The values of the sticking factor for each cluster size, evaluated using the superfluid fraction (black filled circles), the monomer density radial distribution (blue filled triangles), and the total hydrogen density distribution (green crosses). The last one is the same as shown in Fig. 2C. (**C**) Moment of inertia of CH_4_-(*p*H_2_)*_N_* clusters. Red open circles represent experimental values, while blue filled triangles indicate calculated values assuming κ*_N_* = 0.00259(7) for all *N*. The black filled circle at *N* = 12 represents the case where the sticking factor matches the solid value, κ_solid_ = 0.00162.

To analyze details of the superfluid component, we performed PIMC simulations of CH_4_-(*p*H_2_)*_N_* at 0.5 K by the hybrid Monte Carlo algorithm (*36*) using the same potential energy surface adopted in (*35*). Detailed results will be presented in a separate publication. Figure 3A shows the calculated fraction of the superfluid component, which exhibits a drastic increase in this fraction for *N* ≥ 6, and approaches 1.0 around *N* = 10, consistent with earlier reports (*35*).

However, the assumption that the superfluid component contributes negligibly to the moment of inertia in Eq. 3 fails to explain the observed reduction in rotational constants beyond *N* = 10. Further analysis revealed a strong correlation between this decrease and the mass density of the “monomer” component within each (*p*H_2_)*_N_* cluster. In the path integral representation, bosonic exchanges give rise to exchange cycles among particles, which are often described as “cross-linked polymers” in the quantum-classical isomorphism (*10*). The monomer component refers to particles that are not involved in these exchanges and remain isolated from others. The open triangles in Fig. 3A represent the fraction of the monomer component, $\mu_{N}^{M}$, in each cluster derived from the PIMC simulations. Because these monomer components do not participate in bosonic exchanges, it is reasonable to treat them as classical entities, which mainly contribute to the moment of inertia of CH_4_-(*p*H_2_)*_N_*. This finding indicates that Eq. 3 requires modification to account for their influence, as described below

$$I_{\mathrm{tot},N}=I_{CH_{4}}+\kappa_{N}I_{pH_{2},N}^{M}$$ (4)

Here, $I_{pH_{2},N}^{M}$ represents the moment of inertia of the monomer components in the (*p*H_2_)*_N_* clusters. Figure 3B (blue filled triangle) plots the sticking factors obtained using Eq. 4 based on the radial distribution of the monomer components in the (*p*H_2_)*_N_* clusters (details provided in the Supplementary Materials). The resulting sticking factors were approximately κ = 0.002 to 0.003 for all cluster sizes, with a slight increase as the cluster size increased. These values are reasonably larger than the solid-state sticking factor, κ_solid_.

Figure 3C displays the moment of inertia of CH_4_-(*p*H_2_)*_N_* clusters calculated via Eq. 4. Here, instead of applying an independent sticking factor for each cluster size, the observed rotational constants were fitted using a common sticking factor across all cluster sizes. The fitted value of κ*_N_* = 0.00259(7) reproduced the observed rotational constant very well.

On the basis of the above analysis, it becomes evident that a coherent explanation can be derived by attributing the increase in methane’s moment of inertia solely to the density of hydrogen molecules within the para-hydrogen cluster that do not participate in quantum bosonic exchanges. Specifically, the $1-\mu_{N}^{M}$ fraction of CH_4_-(*p*H_2_)*_N_* represented by blue filled triangles in Fig. 3A does not contribute to the moment of inertia due to the quantum bosonic exchanges. It should be noted that this $1-\mu_{N}^{M}$ fraction differs from the standard definition of microscopic superfluidity (*10*), which is represented by the black filled circles in Fig. 3A. The observed *N* dependence in the rotational constant indicates that the moment of inertia of the total system is influenced by the surrounding cluster, even when it exhibits superfluid.

As shown in Fig. 3A, the PIMC simulation revealed an increase in the monomer fraction at *N* = 12. This increase may indicate a magic number effect, as described in (*6*), a phenomenon that occurs when the first shell is fully occupied by 12 *p*H_2_ molecules. Instead of using the value of κ*_N_* = 0.00259, the application of the solid sticking factor, κ_solid_ = 0.00162, specifically for *N* = 12, successfully reproduces the experimental moment of inertia (see the black filled circle in Fig. 3C). This result suggests that (*p*H_2_)_12_ exhibits a more solid-like nature compared to a liquid-like state. As shown in the inset of Fig. 1, the spectral linewidth of *N* = 12 is narrower than that for *N* ≤ 11, further supporting the solid-like nature of (*p*H_2_)_12_, as opposed to the liquid-like nature of *N* ≤ 11 clusters.

**Fig. 4.**
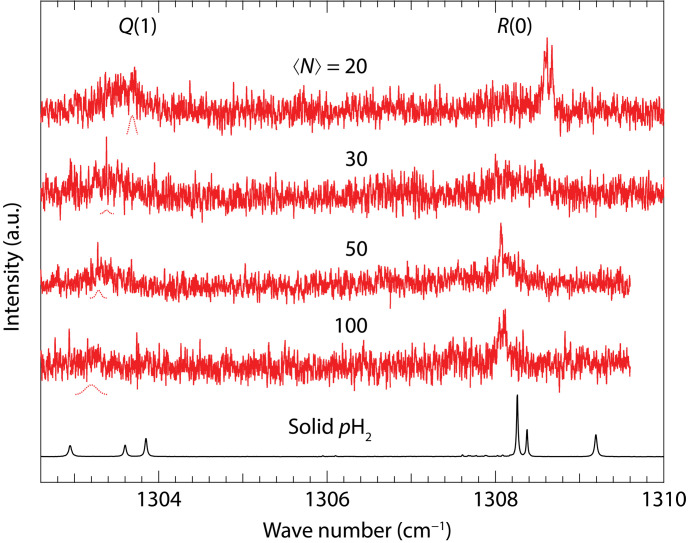
Rotationally resolved ν_4_ spectra of larger CH_4_-(*p*H_2_)*_N_* clusters in helium droplets. Rotationally resolved ν_4_ spectra of CH_4_-(*p*H_2_)*_N_* clusters with ⟨*N*⟩ ≥ 20 in helium droplets (red traces). Dashed lines indicate Gaussian fitted curves for each *Q*(1) transition, added for clarity. The black trace at the bottom shows the spectrum in solid *p*H_2_ crystals (*33*), where multiple peaks appear due to crystal field splitting.

The current signal-to-noise ratio was insufficient to identify peaks corresponding to *N* ≥ 19. Much larger clusters exhibited broadened peaks, as shown in Fig. 4. The size of the clusters shown in Fig. 4 is the average cluster size ⟨*N*⟩ estimated from the known pressure dependence [Poisson distribution (*37*)]. As the cluster size of (*p*H_2_)*_N_* increases further, a red shift is observed in the ν_4_ band of CH_4_. Figure 4 also shows the spectrum of CH_4_ in a *p*H_2_ *hcp* crystal (*33*), recognized as the most stable form of solid *p*H_2_. It should be noted that the spectral peak positions of these larger CH_4_-(*p*H_2_)*_N_* clusters do not align with the peaks observed in the *hcp* crystal structure (*33*). This discrepancy suggests that the surrounding *p*H_2_ clusters differ from the stable crystal structure, even though larger clusters may transition toward a solid state due to nucleation. The data in Fig. 4 imply that larger CH_4_-(*p*H_2_)*_N_* clusters may retain a liquid-like state, highlighting the need for further analysis.

## DISCUSSION

In this study, we have provided experimental evidence supporting the microscopic superfluidity of *p*H_2_ clusters at 0.4 K, as observed through the rotational transitions of CH_4_-(*p*H_2_)*_N_*. For cluster sizes up to *N* ≤ 18, we observed gradual reduction in the rotational constant of CH_4_-(*p*H_2_)*_N_* as additional *p*H_2_ molecules were incorporated. This cluster size dependence is attributed to interactions of CH_4_ with neighboring (*p*H_2_)*_N_* molecules that do not engage in quantum bosonic exchanges. Our PIMC simulations further revealed that more than 60% of the (*p*H_2_)*_N_* clusters with *N* ≥ 5 participate in quantum bosonic exchanges, confirming the quantum nature of these systems. The effects of the surrounding helium droplet were not explicitly included in the present PIMC simulation when evaluating the monomer components; however, these effects are expected to have minor contributions compared to the dominant interactions between CH_4_ and the surrounding (*p*H_2_)*_N_* cluster (*21*). Future studies will address the influence of helium droplets to provide a more comprehensive understanding of these interactions as well as finite temperature effects.

Moreover, the spectra of substantially larger (*p*H_2_)*_N_* clusters, with *N* approximating 100, continued to deviate from those observed in solid *p*H_2_, suggesting that even at these larger sizes, the CH_4_-(*p*H_2_)*_N_* clusters likely retain a liquid-like state. These findings demonstrate the unique capability of CH_4_ as a probe for superfluidity. Its light mass, spherical symmetry, and weak intermolecular interactions minimize disruptions to the surrounding environment, thereby preserving the integrity of the superfluid state.

The successful application of this technique opens up exciting possibilities for observing molecular superfluidity in even larger hydrogen clusters, paving the way for deeper explorations of quantum phenomena in finite molecular systems.

## MATERIALS AND METHODS

### Experimental design

The experimental apparatus used in this study wats similar to that described in (*26*). In brief, helium droplets were generated by continuously expanding low-temperature, high-pressure helium gas into vacuum through a nozzle with a 5-μm aperture (*38*). The stagnation pressure of the helium gas was maintained at 2 MPa. The average size of the droplets, *N*_He_, was regulated by the nozzle temperature *T*_0_, setting it to *T*_0_ = 11 K for *N*_He_ = 1.5 × 10^4^ and *T*_0_ = 16 K for *N*_He_ = 2.1 × 10^3^, respectively (*39*). Following a skimmer, the droplet beam passed through two pickup cells. The first cell contained methane vapor, whose pressure was adjusted to maximize the monomer peak of methane in helium droplets. The second pickup cell, located 5 cm downstream (see fig. S5) was filled with *p*H_2_ vapor, in which helium droplets with CH_4_ pick up multiple *p*H_2_ molecules to form CH_4_-(*p*H_2_)*_N_* clusters. The typical pressure in each cell was 10^−3^ and 10^−2^ Pa for CH_4_ and *p*H_2_, respectively. The pressure deviation was ∼5%. The *p*H_2_ gas was obtained by the catalytic conversion method with hydrous ferric oxide kept at 13.8 K (*40*).

A single CH_4_ molecule inside each droplet was excited using counter-propagating infrared radiation at 7 μm obtained from a quantum cascade laser (Daylight Solutions, TLS-21077-MHF). The laser frequency was calibrated by gaseous CH_4_ absorption along with a homemade etalon to further interpolate the frequency to an absolute certainty of better than 10 MHz. The absorption of CH_4_ in helium droplets was detected by the depletion of mass signals [*m*/*z* (mass/charge ratio) ≥ 6] using a quadrupole mass spectrometer with an electron beam ionizer.
