# Supplementary material for: Exploring molecular superfluidity in hydrogen clusters
Source: Sci Adv. 2025 Feb 21;11(8):eadu1093. doi: 10.1126/sciadv.adu1093 (PMC11844719; doi:10.1126/sciadv.adu1093)
Supplement: Supplementary file 1 — Supplementary Text Figs. S1 to S5 Table S1 [file sciadv.adu1093_sm.pdf]

Science Advances
AAAS

Supplementary Materials for

Exploring molecular superfluidity in hydrogen clusters

Hatsuki Otani *et al.*

Corresponding author: Susumu Kuma, susumu.kuma@riken.jp; Takamasa Momose, momose@chem.ubc.ca

Sci. Adv. **11**, eadu1093 (2025)
DOI: 10.1126/sciadv.adu1093

This PDF file includes:

Supplementary Text
Figs. S1 to S5
Table S1

Supplementary Text

Results of PIMC simulations of CH_4–($p$$H_2$)$_N$: $p$$H_2$ distribution

The radial distributions of $p$$H_2$ molecules around a CH_4 molecule were calculated using the PIMC method. In these simulations, the methane molecule was approximated as a sphere surrounded by varying numbers of $p$$H_2$ molecules at T = 0.5 K.

Figure S1 illustrates the radial distribution of $p$$H_2$ molecules relative to the central CH_4 molecule, equivalent to the total $p$$H_2$ number density. An almost constant increase in the peak around 4.2 Å was observed for cluster size up to N = 12, with a slight shift of the peak toward longer distances for N = 13–18. These distributions suggest that the $p$$H_2$ molecules form a first solvation shell around CH_4 at these cluster sizes. Notably, no distinct peak corresponding to a second solvation shell was observed, even for N > 13.

Figure S2 presents the radial distribution of monomer components calculated via PIMC simulations. Unlike Fig. S1, these distributions exhibit non-uniform changes with cluster size N, reflecting the complex characteristics of bosonic exchange. For N = 2–5, the peak of the monomer density distribution increases almost uniformly around 4.2 Å. However, for 6 ≤ N ≤ 10, the peak height exhibits minimal variation, suggesting that bosonic permutation exchanges become more prominent once a hexamer cycle forms. At N = 11, the peak height increases, and at N = 12, it becomes noticeably larger, indicating a "magic number" effect when the first solvation shell is fully occupied by twelve $p$$H_2$ molecules. For N > 12, the peak height decreases compared to N = 12 and shifts slightly toward longer distances, consistent with enhanced bosonic permutation exchanges.

To examine the geometric structure of bosonic exchanges, the angular dependence of the total $p$$H_2$ distribution for N = 12 and N = 13 was compared with the density profiles of $p$$H_2$ molecules involved in bosonic permutation exchanges. Figure S3 shows the angular dependence of the total $p$$H_2$ distribution for N = 12 (A) and N = 13 (B) as cross-sectional views, with the methane molecule positioned at the origin. As expected, the total density profiles exhibit isotropic distributions relative to the central CH_4 molecule. In contrast, Fig. S4 displays the density profiles of $p$$H_2$ molecules involved in bosonic permutation exchanges within the hexamer cycle for N = 12 (A) and N = 13 (B). In these figures, the y-axis corresponds to the direction yielding the maximum eigenvalue of the hexamer's moment of inertia tensor. The anisotropy in Fig. S4 indicates a ring-like exchange density, consistent with findings reported by Mak *et al.* (*32*). These figures reveal that the $p$$H_2$ molecules along the polar direction (y-axis) are excluded from the ring-shaped exchange structure, resulting in a concentration of monomer density along the y-axis.

Finally, the calculated moments of inertia for both the total and monomer $p$$H_2$ distributions, $I_{p\mathrm{H_2},N}$ and $I^{\mathrm{M}}_{p\mathrm{H_2},N}$, are listed in Table S1. These values were used to reproduce the experimental rotational constants by incorporating the sticking factors discussed in the main text.

Experimental configuration of the pickup cells

Figure S5 shows the layout of the pickup cells along the beam path. The pressures within the cells were controlled using leak valves installed in the gas lines outside of the vacuum chamber.

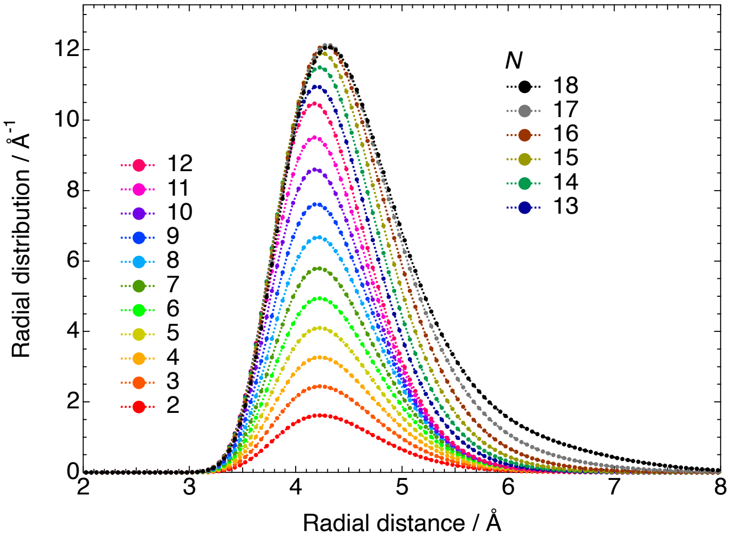

Fig. S1. Radial distribution of *p*H_2 density around CH_4.

Radial distribution of the total *p*H_2 density around the CH_4 molecule for different *p*H_2 cluster sizes *N* (*N* = 2–18).

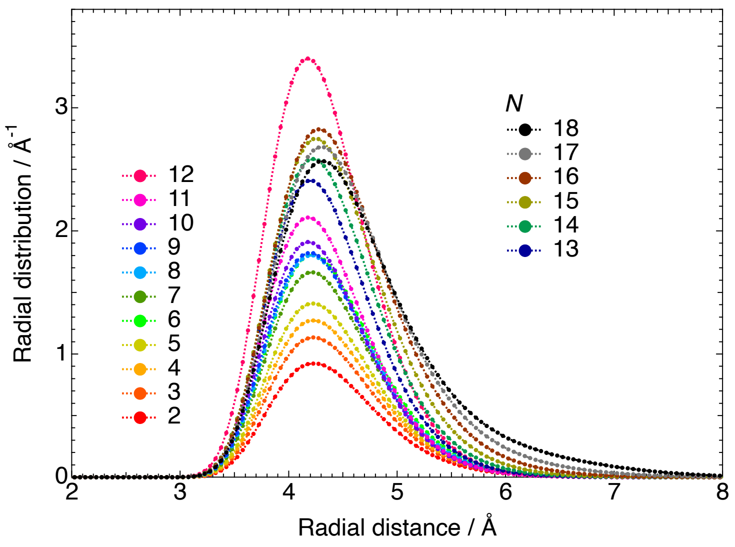

Fig. S2. Radial distribution of monomer pH$_2$ density around CH$_4$.

Radial distribution of the monomer pH$_2$ density (non-exchanging) around the CH$_4$ molecule for different pH$_2$ cluster sizes N ($N = 2$–18).

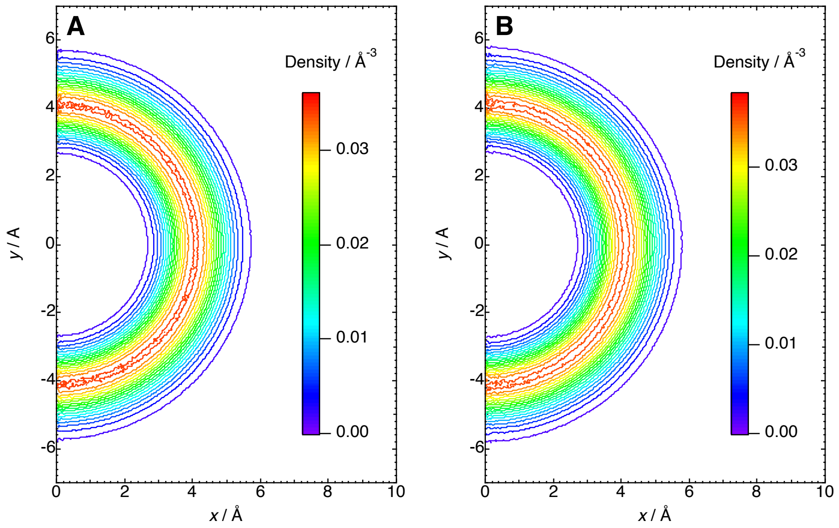

Fig. S3. Cross-sectional views of pH$_2$ density profiles for N=12 and N=13.

Two-dimensional cross-sectional views of the total pH$_2$ density profiles for cluster sizes N = 12 (**A**) and N =13 (**B**). The CH_4 molecule is positioned at the origin. The contour color represents the density, with the maximum observed around 4.2 Å.

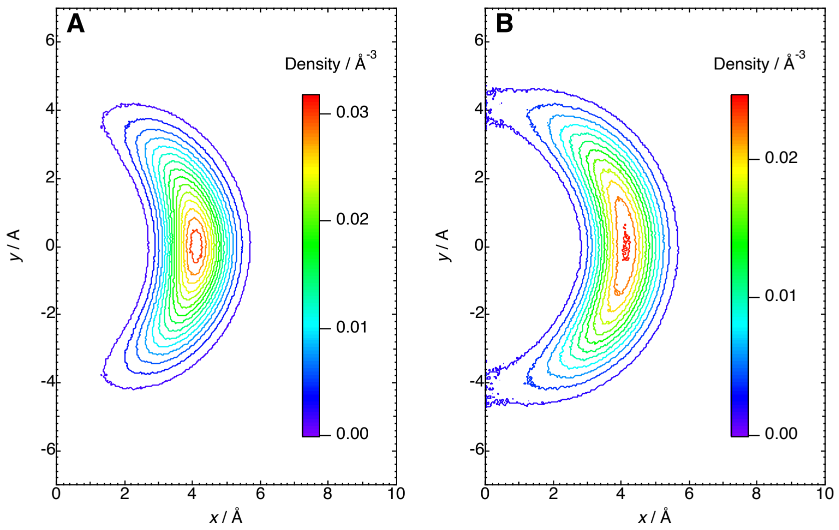

Fig. S4. Cross-sectional views of pH$_2$ density profiles in bosonic permutation cycles.

Two-dimensional cross-sectional views of the pH$_2$ density profiles involved in the bosonic permutation cycle of the hexamer for the cluster sizes $N = 12$ (**A**) and $N = 13$ (**B**). The CH_4 molecule is positioned at the origin.

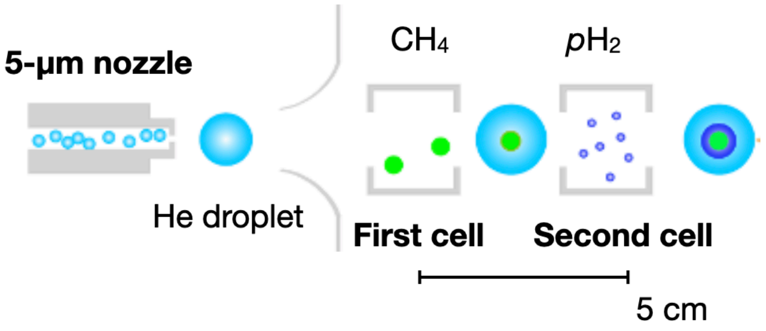

Fig. S5. Experimental layout of pickup cells along the He droplet beam path.
Schematic representation of the experimental layout showing the pickup cells positioned along the helium droplet beam path.

Table S1. Calculated moments of inertia for pH$_2$ clusters.

Calculated moments of inertia for pH$_2$ clusters: Total distribution ($I_{p\mathrm{H}_2,N}$) and monomer distribution ($I^{\mathrm{M}}_{p\mathrm{H}_2,N}$).

N	$I_{p\mathrm{H}_2,N}$ / uÅ^2	$I^{\mathrm{M}}_{p\mathrm{H}_2,N}$ / uÅ^2
2	41.17	30.39
3	59.09	36.99
4	80.25	41.27
5	103.60	45.37
6	127.44	57.80
7	157.16	52.71
8	186.60	56.37
9	218.09	55.83
10	248.27	57.04
11	275.64	62.53
12	301.12	99.32
13	332.89	74.21
14	364.42	83.06
15	398.32	93.27
16	435.97	103.04
17	479.40	107.34
18	529.71	114.14
